# Gut Microbiota Associations with Metabolic Health and Obesity Status in Older Adults

**DOI:** 10.3390/nu12082364

**Published:** 2020-08-07

**Authors:** Xiaozhong Zhong, Janas M. Harrington, Seán R. Millar, Ivan J. Perry, Paul W. O’Toole, Catherine M. Phillips

**Affiliations:** 1 National Engineering Laboratory for Cereal Fermentation Technology, Jiangnan University, Wuxi 214122, China; xiaozhong_zhong@jiangnan.edu.cn; 2 School of Microbiology and APC Microbiome Ireland, University College Cork, Cork, Ireland; pwotoole@ucc.ie; 3 HRB Centre for Health and Diet Research, School of Public Health, University College Cork, Cork, Ireland; j.harrington@ucc.ie (J.M.H.); s.millar@ucc.ie (S.R.M.); i.perry@ucc.ie (I.J.P.); 4 School of Public Health, Physiotherapy, and Sports Science, University College Dublin, Dublin 4, Ireland

**Keywords:** gut microbiota, metabolic syndrome, host-microbiota interactions, metabolically healthy obesity, obesity phenotypes, older adults

## Abstract

Emerging evidence links the gut microbiota with several chronic diseases. However, the relationships between metabolic syndrome (MetS), obesity and the gut microbiome are inconsistent. This study aimed to investigate associations between gut microbiota composition and diversity and metabolic health status in older adults (*n* = 382; median age = 69.91 [± 5 years], male = 50.79%) with and without obesity. Gut microbiome composition was determined by sequencing 16S rRNA gene amplicons. Results showed that alpha diversity and richness, as indicated by the Chao1 index (*p* = 0.038), phylogenetic diversity (*p* = 0.003) and observed species (*p* = 0.038) were higher among the metabolically healthy non-obese (MHNO) individuals compared to their metabolically unhealthy non-obese (MUNO) counterparts. Beta diversity analysis revealed distinct differences between the MHNO and MUNO individuals on the phylogenetic distance scale (R^2^ = 0.007, *p* = 0.004). The main genera contributing to the gut composition among the non-obese individuals were *Prevotella*, unclassified Lachnospiraceae, and unclassified Ruminococcaceae. *Prevotella*, *Blautia*, *Bacteroides*, and unclassified Ruminococcaceae mainly contributed to the variation among the obese individuals. Co-occurrence network analysis displayed different modules pattern among different metabolic groups and revealed groups of microbes significantly correlated with individual metabolic health markers. These findings confirm relationships between metabolic health status and gut microbiota composition particularly, among non-obese older adults.

## 1. Introduction

Metabolic syndrome (MetS) is characterized by a clustering of metabolic abnormalities and risk factors for cardiovascular disease (CVD) including central obesity, hypertension, dyslipidemia and elevated fasting glucose [1,2]. The increasing incidence of MetS is associated with high morbidity and mortality and concomitant economic and social burden [3]. Thus, MetS represents a significant public health concern requiring more effective preventative and therapeutic strategies. The etiology of MetS is not fully understood. Obesity plays a significant role; however, obesity is a heterogeneous condition and not all individuals with obesity display MetS features [4]. It has been recognized that some individuals with obesity have preserved metabolic function generally defined as an absence of MetS and may be regarded as metabolically healthy obese (MHO). Similarly, not all individuals without obesity are metabolically healthy; thus a spectrum of metabolic health phenotypes exist from metabolically healthy non-obese (MHNO) to metabolically unhealthy obese (MUO) [5].

Gut microbiota is regarded as a central factor co-varying many chronic diseases including CVD, type 2 diabetes mellitus (T2DM), and MetS [6,7]. Subsequently, specific microbiota-based biomarkers associated with these conditions have been identified [8,9,10]. Although previous studies have reported associations between gut microbiota and metabolic disorders and obesity, no consistent gut microbiota signatures have been identified for MetS. Some previous studies have reported the relative abundance of Bacteroidetes members as being lower, and the proportions of Firmicutes members higher, in the gut microbiota of obese subjects [11,12]. On the contrary, increased relative abundance of Bacteroidetes members have been observed in overweight and obese subjects [13,14].

Treatments targeting gut microbiota directly such as probiotics, prebiotics, [15,16], and faecal transplantation [17] or indirectly via bariatric surgery [18] can positively influence metabolic health. The heterogeneity of human microbiota, which is influenced by sex, age, race and geographical location amongst other factors [19,20,21], is becoming more apparent. It is well documented that aging is associated with alterations in the gut microbiome [22,23,24]. Thus, better understanding of the gut microbiota heterogeneity according to metabolic health and weight status, particularly among older adults, is warranted, with a view to improving our understanding of the role of gut microbiota in MetS, obesity-related disease and healthy ageing and for developing novel preventative and treatment strategies. To our knowledge, no comparative data on gut microbiota according to metabolic health phenotype, such as metabolically healthy and unhealthy obese and non-obese exist. Therefore, the aim of this study was to examine associations between metabolic health phenotypes and gut microbiota composition and diversity in older male and female adults.

## 2. Materials and Methods

### 2.1. Study Participants

Participants were recruited to the Mitchelstown Cohort Rescreen (MCR) Study, which was a follow-up study to the 2010 Cork and Kerry Diabetes and Heart Disease Study [25]. Of the 2047 participants that took part in the initial phase of the study, 1881 were eligible to participate in the follow-up phase; the remaining 166 participants from the initial phase of the study had either passed away between completion of the Cork and Kerry Diabetes and Heart Disease Study and the start of the MCR Study, or were deemed to be medically ineligible, as determined by their contact General Practitioner (GP). Between November 2015 and May 2017, 1378 participants returned for the rescreen study (73% response rate). All participants were between the ages of 55 and 74 years. The MCR sample is a population-representative random sample, recruited through the Living Health Clinic, a large primary care center in Ireland. Of the 1378 participants, 435 participants (31.6%), without selecting for any particular health or disease status, provided stool samples for gut microbiota analysis. After exclusion of the subjects who used antibiotics in the previous 4 weeks, 382 participants (27.7%) were included in the current analysis.

All subjects gave their informed consent for inclusion before they participated in the study. The study was conducted in accordance with the Declaration of Helsinki, and the protocol was approved by the Ethics Committee of the Clinical Research Ethics Committee of University College Cork (Project identification code ECM 4 (nnn) 07/07/15). The MCR Study is General Data Protection Regulation (GDPR) compliant. All procedures and measurements were conducted by trained research staff according to study-specific standard operating procedures.

### 2.2. Measurements

#### 2.2.1. Demographic and Covariate Information

Blood samples were taken following an overnight fast. Fasting plasma glucose (FPG), glycated haemoglobin A_1c_ (HbA_1c_), serum total cholesterol, HDL cholesterol (HDL-C), LDL cholesterol (LDL-C) and triglyceride levels were measured by Cork University Hospital Biochemistry Laboratory using fresh blood samples. Resting systolic blood pressure (SBP) and diastolic blood pressure (DBP) were measured by standard auscultatory methods when the subjects were relaxed. Body composition was measured via bioelectrical impedance (BIA) (Tanita MC 780_MA Body Composition Analyser, Tanita, Amsterdam, Netherlands). Height (cm) and weight (kg) were measured without footwear or heavy outer clothes using a portable stadiometer (Seca, Leicester, United Kingdom) and portable electronic scales (Tanita, Amsterdam, Netherlands), respectively. Body mass index (BMI) was calculated using weight and height squared (kg/m^2^). Data on covariates, including age, sex, disease status (CVD and T2DM), medication use (blood pressure, cholesterol lowering, diabetes medications and antibiotics) and lifestyle factors (smoking status and alcohol consumption) were obtained by asking each participant to complete a clinical report form and a computer-assisted personal interview general health questionnaire. The presence of CVD was based on diagnosis of any one of the following conditions: hypertension, high cholesterol, heart attack (including coronary thrombosis or myocardial infarction), heart failure, angina, aortic aneurysm, stroke, narrow leg arteries or any other heart condition reported by the participant.

In addition, participants also completed a standard validated Food Frequency Questionnaire (FFQ), from which the Dietary Approaches to Stop Hypertension (DASH) diet quality scores were derived [26].

#### 2.2.2. Classification of MetS and Metabolic Health Phenotypes

MetS was defined according to the revised National Cholesterol Education Panel Adult Treatment Panel III (NCEP ATP III) guidelines which classifies MetS as three or more of the following risk factors [27]: (1) obesity classified as waist circumference ≥ 102 cm in men or ≥ 88 cm in women, (2) dysglycemia defined as a FPG level ≥ 5.6 mmol/L, (3) fasting hypertriglyceridemia (triglycerides > 1.7 mmol/L), (4) low HDL-C level (HDL-C < 1.03 mmol/L in men or < 1.29 mmol/L in women), (5) high blood pressure (SBP ≥ 130 mmHg or DBP ≥ 85 mmHg or on blood pressure medications). Metabolic health status was defined according to obesity (BMI ≥ 30 kg/m^2^) with or without the presence of MetS [28], generating the following groups; metabolically healthy non-obese (MHNO), metabolically healthy obese (MHO), metabolically unhealthy non-obese (MUNO), and metabolically unhealthy obese (MUO) subjects.

### 2.3. Gut Microbiota Analyses

Stool collection was performed by the participant at their home. Participants were provided with a container to collect the whole bowel motion. They were advised that the stool sample should be as fresh as possible, ideally produced on the morning of their visit to the clinic where the samples were stored at −80 °C until extraction. If this was not possible, a sample from the previous night (within 12 h of their visit to the clinic) was permitted. Microbiota analysis was performed as previously described [29]. The composition of the gut microbiome in faecal samples was determined by 16S rRNA gene sequencing. The V3-V4 region of the 16S rRNA gene was amplified and sequenced on an Illumina MiSeq 2 × 250 bp using the following primers: 341F (5′-CCTACGGGNGGCWGCAG-3′) and 805R (5′-GACTACHVGGGTATCTAATCC-3′). Flash was used to join overlapping paired reads and exclude reads that have more than 25% incorrect bases in the region of overlap. Quality control was then carried using Quantitative Insights Into Microbial Ecology (QIIME) 1.9.0. Reads were then summarized to Operational Taxonomic Units (OTUs), with a 97% identity threshold and chimeric sequences removed using USEARCH64. In order to calculate alpha (within-participants) and beta (between-participants) diversity, the complete OTU count Table was rarefied to 10,000 sequences. Alpha and beta diversity metrics were calculated for the rarefied Tables using QIIME 1.9.0. The representative OTU sequences were assigned taxonomy by Mothur 1.36.1 using the RDP database and the RDP method [29].

### 2.4. Statistical Analyses

Statistical analyses were performed using the R statistical package (3.5.1). Normality of variables was assessed using the Shapiro-Wilk test. Those variables that did not meet normality were analyzed using a Mann–Whitney U test. A Chi-Square test was used to compare differences for categorical variables. Multiple linear regression analyses were performed to assess the associations of metabolic health markers (MetS components) with alpha diversity and abundant taxonomy (relative abundance > 0.1%) following adjustment for potential confounders, such as age, sex, disease status (CVD and/or T2DM), medication use (blood pressure, cholesterol lowering, diabetes medications) and lifestyle factors (DASH score, smoking status and alcohol consumption). An analysis of variance (ANOVA) test was used to investigate associations between the metabolic health markers and alpha diversity and relative abundance of taxonomy in linear models. Differences in beta diversity (principal coordinates analysis, PCoA) were investigated by permutational multivariate analysis of variance based on distance matrices using *adonis* in R *vegan* package, following adjustment for potential confounders. Statistical significance was initially set at an alpha level of 0.05. Differential abundance at phylum, family and genus level between MHNO and MUNO, MHO and MUO groups was identified using *DESeq2* R package. In multivariate analyses, *p* values were corrected with the Benjamini-Hochberg false discovery rate (FDR < 0.05).

### 2.5. Co-Occurrence Network Analysis

OTU read counts were normalized by variance stabilizing transformation in the *DESeq2* R package. Co-occurrence network based on the normalized OTU was constructed using the weighted gene co-expression network analysis (WGCNA) R package [30]. Briefly, we set deepSplit = 2 and minModuleSize = 10 as parameters for the module identification using dynamic tree cut. The relationship between modules and metabolic trait was calculated using the Spearman correlation. Visualization of the network was performed using the *igraph* R package.

## 3. Results

### 3.1. Participant Characteristics

Samples from 382 participants (median age = 69.91 (±5 years), male = 50.79%, female = 49.21%) were analyzed, including 191 individuals with MHNO, 61 with MUNO, 66 with MHO and 64 MUO subjects (Table 1). Age, sex, total cholesterol, smoking and alcohol consumption were not significantly different among groups. Anthropometric (BMI, waist circumference) and clinical parameters including SBP, HbA_1c_, fasting glucose, VLDL-C and triglyceride concentrations were higher and HDL-C concentrations were lower among the MUNO and MUO groups compared to their metabolically healthy counterparts (MHNO and MHO). Although dietary differences (dietary quality and macronutrient composition) were observed when all four groups were compared, no differences were noted within the non-obese (MHNO vs. MUNO) or obese groups (MHO vs. MUO).

### 3.2. Metabolic Health Phenotype Associations with Gut Microbiota Alpha Diversity

**Figure 1:**
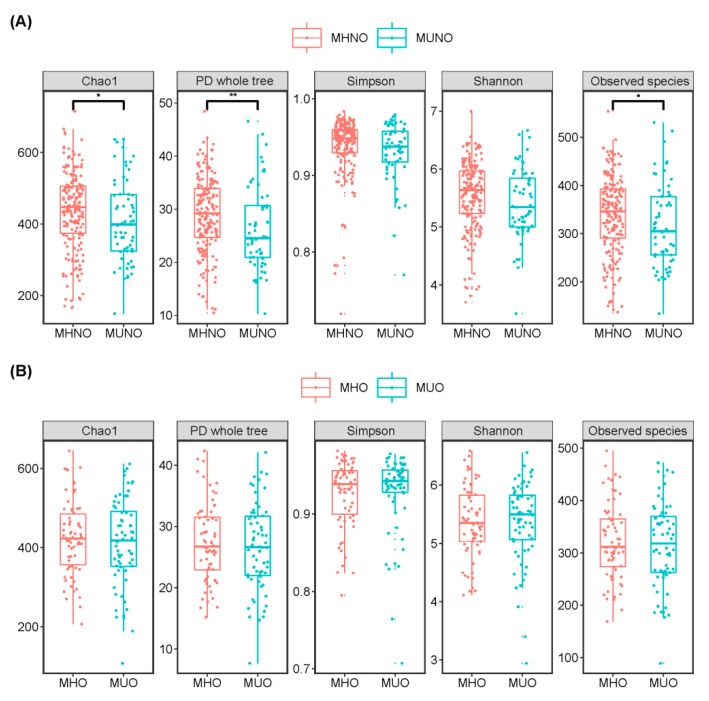
Alpha diversity comparisons between (**A**) MHNO and MUNO individuals, (**B**) MHO and MUO individuals. *p*-values were calculated using Mann-Whitney U tests for unpaired groups. Only the comparisons with false discovery rate-adjusted *p* values < 0.05 are presented. * *p* < 0.05, ** *p* < 0.01.

Higher gut microbiota alpha diversity was observed among the MHNO participants, relative to their MUNO counterparts. This was attributed to the significantly higher richness and diversity as indicated by the Chao1 index (*p* = 0.038), phylogenetic diversity (*p* = 0.003) and observed species (*p* = 0.038) among the MHNO individuals (Figure 1A). No significant difference in gut microbiota evenness, as assessed by the Shannon and Simpson indexes, was observed between the MHNO and MUNO groups. No differences were observed in any measure of alpha diversity between the MHO and MUO groups (Figure 1B).

Associations between individual metabolic health markers and microbiome alpha diversities were assessed by multivariate linear regression (Supplementary Table S1). Total cholesterol and LDL-C showed significant (FDR < 0.05) positive relationships with Chao1, Shannon and phylogenetic diversity adjusted for age, sex, disease status and lifestyle factors. Triglyceride and VLDL-C concentrations were negatively (FDR < 0.05) associated with Chao1 index. No significant associations were observed between other metabolic health markers and alpha diversity.

### 3.3. Metabolic Health Phenotype Associations with Gut Microbiota and Beta Diversity

Beta diversity (PCoA) analysis showed that gut microbiota of MHNO individuals was significantly (R^2^ = 0.007, *p* = 0.004) distinct from that of the MUNO subjects on phylogenetic distance scale (unweighted UniFrac, Figure S1). However, when considering the abundance of OTUs, no significant difference was observed between the two groups (Figure S1). In addition, beta diversity of the gut microbiota was not significantly different between the obese subjects (MHO vs. MUO) (Figure S2). Moreover, we investigated whether bacterial community was correlated with individual metabolic health markers by PERMANOVA. In the fully adjusted model, none of the metabolic health markers were associated with inter-individual distance of microbial composition (Bray–Curtis distance) at a FDR of <0.05 (Table S2).

**Figure 2:**
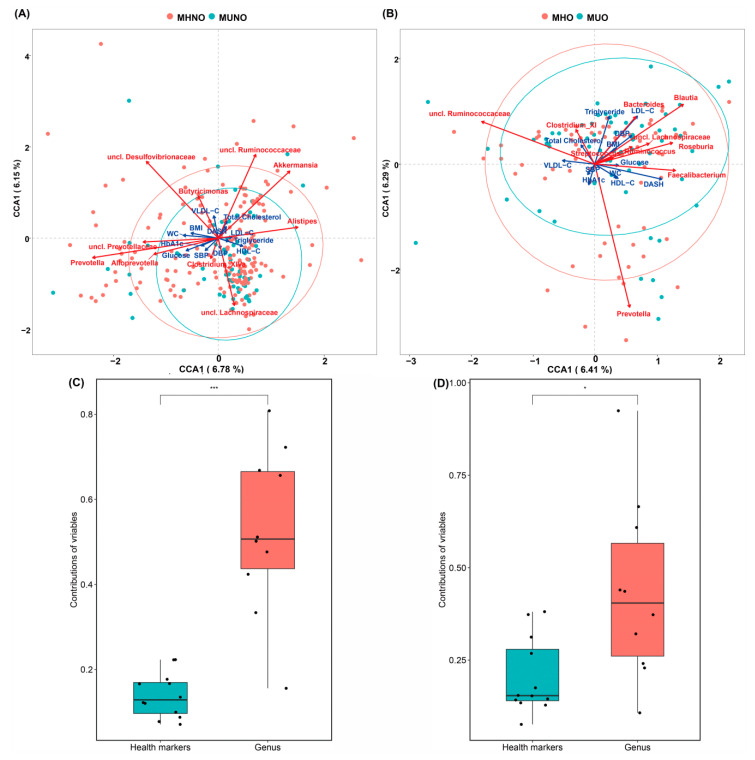
Microbial community variation in the MCR cohort. (**A**) MHNO vs. MUNO, (**B**) MHO vs. MUO, (**C**) the contribution of variables to the variance of MHNO and MUNO bacterial community, (**D**) the contribution of variables to the variance of MHO and MUO bacterial community. Top contributors of genus to the community variation as determined by redundancy analysis on scaled OTUs abundances (red arrows); metabolic health markers contribute to microbiome community variation (blue arrows). Arrows scaled to contribution. * *p* < 0.05, *** *p* < 0.001.

We assessed how gut microbiota community composition and metabolic health markers covaried with beta diversity differences in gut microbiota in this cohort. Based on constrained correspondence analysis of OTUs and genus-level community composition, we found that the main genera contributing to the gut microbiota compositional differences were *Prevotella*, unclassified Lachnospiraceae genus, *Akkermansia* and unclassified Ruminococcaceae in MHNO and MUNO groups (Figure 2A). *Prevotella*, *Blautia*, *Bacteroides*, and unclassified Ruminococcaceae mainly contributed to the variation of MHO and MUO communities (Figure 2B). The top 10 contributors at genus level in communities of participants without obesity were different from those obese individuals.

In terms of the co-variation of metabolic health markers with the microbiota variation, vectoral differences of HbA_1c_, glucose, BMI, WC, and SBP values showed a similar direction of association with *Prevotella* genus, while HDL-C and triglyceride formed a directional trend with *Alistipes* in MHNO and MUNO groups. For MHO and MUO groups, vectors of LDL−C, triglycerides, DBP and BMI value gradients showed a similar direction of association to *Bacteroides* and *Blautia*; glucose, WC, HDL-C coincided with genus *Faecalibacterium*; HbA_1c_ and SBP formed acute vector with *Prevotella*. However, among the associations we detected, the contribution of the individual metabolic health markers was significantly lower than those of the genus factors (Figure 2C,D).

### 3.4. Fine-Detail Taxonomic Composition of Gut Microbiota

We further interrogated the composition of gut microbiota at different taxonomic levels (Figures S3 and S4). Sequences were sorted into 6527 OTUs (≥97% identity). Of these OTUs, 490 were present in at least 20% of the samples. Fecal microbial communities displayed a typical Western diversity profile dominated by phyla Firmicutes (mean = 76.44%, range = 23.43–98.48%) and Bacteroidetes (mean = 15.51 %, range = 0.03–58.29%) (Figure S3). Overall, 15 phyla were determined in the cohort fecal microbial communities, but no significantly different phylum was observed in metabolic health phenotype groups (Table S3). The most abundant families (90% of total sequences) belong to Lachnospiraceae (mean = 32.24%, range = 4.37–80.05%), Ruminococcaceae (mean = 28.51%, range = 0.14–55.63%), Prevotellaceae (mean = 6.87%, range = 0.00–57.67%), and Bacteroidaceae (mean = 5.70%, range = 0.01–37.69%) (Figure S4). Relative abundance of Lachnospiraceae was significantly higher among the MUNO individuals relative to the MHNO individuals (Table S4). At the genus level, 72 main genera were detected in at least 20% of the samples. Faecalibacterium (mean = 11.20%) was the most abundant phylotype across 382 MCR samples ranging from 0 to 42.27%, followed by unclassified Ruminococcaceae (mean = 11.08%, range = 0.06–42.26%), unclassified Lachnospiraceae (mean = 9.67%, range = 1.17–24.77%), Blautia (mean = 6.85%, range = 0.41–30.64%) and Prevotella (mean = 6.68%, range = 0–56.80%) (Figure S5). No genus differences were observed between the obese metabolic health groups (Table S5).

### 3.5. Gut Microbiota Taxonomy and Metabolic Health Marker Associations

**Figure 3:**
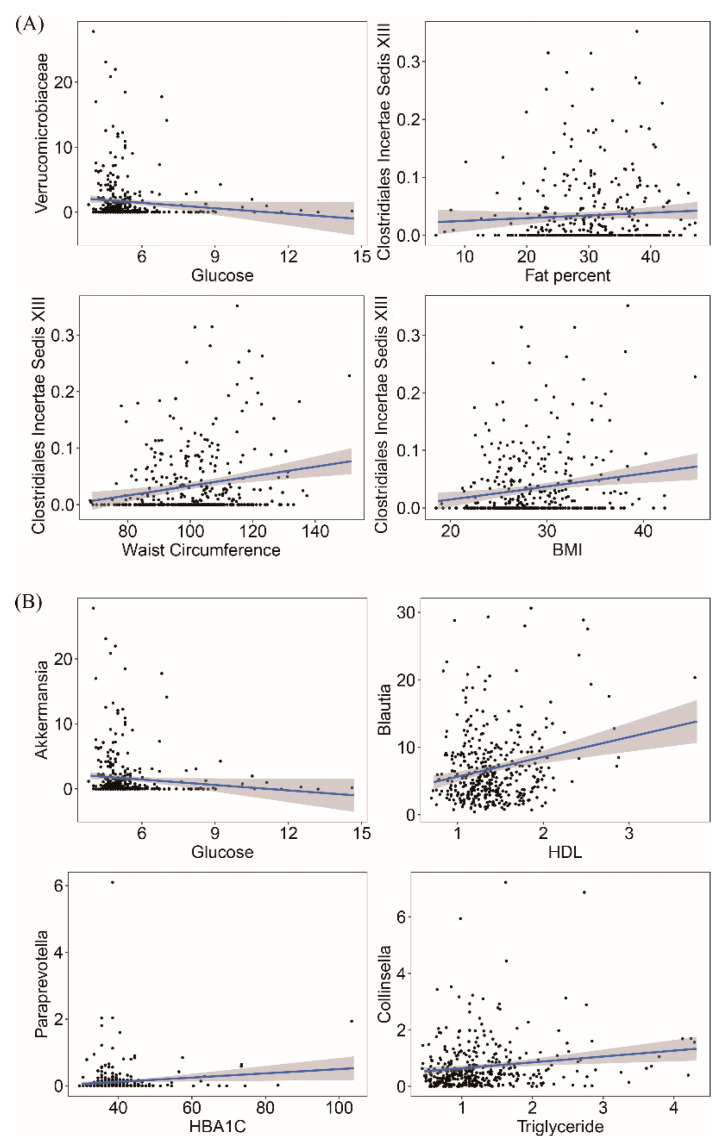
Significant associations between metabolic health markers and taxonomy. Examples of linear regression of metabolic health markers at family level (**A**) and genus level (**B**). Permutational multivariate analysis adjusted for age, sex, disease status (CVD and T2DM), medication use (blood pressure, cholesterol lowering and diabetes medication), lifestyle factors (DASH score, smoking status and alcohol consumption).

We next assessed the associations of the gut microbiota with metabolic health markers. At the phylum level, only Verrucomicrobia negatively correlated with fasting glucose (R^2^ = 0.021, FDR = 0.009) (Table S6). At the family level, 6 significant associations were observed between 12 metabolic health markers and 29 main families (Table S7). For example, Verrucomicrobiaceae was negatively associated with FPG while positive associations of Clostridiales Incertae Sedis XIII with BMI, WC and fat percent were observed (Figure 3A). Figure S5 showed the relative abundance distribution at genus level according to the MetS groups. We found 72 main genera in at least 20% of the samples (supplementary notes). Of these genera, 12 genera were significantly associated with metabolic health markers (Table S8). For instance, *Akkermansia* was negatively correlated with FPG; *Blautia*, *Paraprevotella* and *Collinsella* were positively associated with HDL-C, HbA_1c_ and triglycerides and *Anaerostipes* was negatively correlated with BMI and WC (Figure 3B and Table S8).

### 3.6. OTU Co-Occurrence Network and Metabolic Health Marker Associations

**Figure 4:**
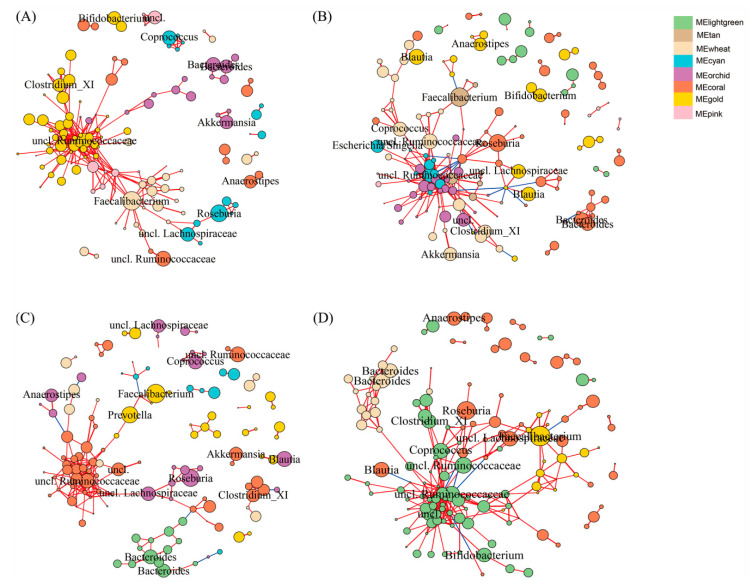
OTU co-occurrence network according to metabolic health phenotype. MHNO (**A**), MUNO (**B**), MHO (**C**) and MUO (**D**). OTUs (nodes) are colored according to WGCNA module colors. Positive correlations are marked by blue edges correspond and negative correlations are marked by red edges. Edges width and length are scaled to the correlation coefficient. Any resulting correlations with *p* value *≥* 0.05 and abs(r) < 0.6 were removed. Circle size indicates the normalized relative abundance of OTU, and the OTUs with relative abundance > 1% are marked at genus level.

**Figure 5:**
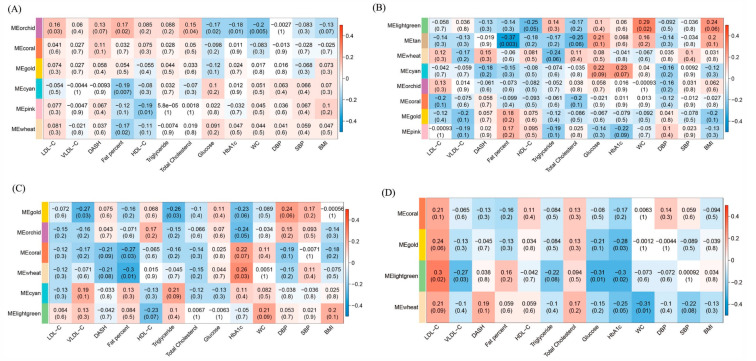
Module–metabolic health markers associations are shown according to metabolic health phenotype. MHNO (**A**), MUNO (**B**), MHO (**C**) and MUO (**D**). Each cell of the matrix contains the coefficient between one OTU module and a health marker, and the corresponding *p* value.

To identify the microbial communities associated with MetS, we clustered the 490 main OTUs (relative abundance > 0.1%) into modules (containing > 10 OTUs) according to the co-occurrence of their relative abundance depending on metabolic health phenotype (Figure 4). The associations of each module with metabolic health markers are shown in Figure 4. Gut microbiota of MHNO individuals were divided into six modules (Figure 4A). Few significant correlations were found in the MHNO participants. The module labeled MEorchid which is dominated by *Bacteroides* and *Akkermansia* was positively associated with LDL-C, fat percent and total cholesterol, and was negatively associated with glucose HbA_1c_ and WC (Figure 5A). The module MEcyan containing *Roseburia* and MEwheat containing *Faecalibacterium* were negatively correlated with fat percent (Figure 5A). Among the MUNO individuals, OTUs were clustered into eight modules (Figure 4B). The MElightgreen module, which contains less abundant OTUs (relative abundance < 1%) from *Ruminococcus* and *Blautia* sp., was positively correlated with WC counts (Figure 5B). The *Faecalibacterium* dominated module (MEtan) was also negatively correlated with fat percent (Figure 5B).

Regarding the obese individuals, the MHO community was divided into six modules (Figure 4C). The *Faecalibacterium* and *Prevotella* dominated module (MEgold) displayed negative correlations with VLDL-C and triglyceride (Figure 5C). The unclassified Ruminococcaceae, Clostridium XI, and *Akkermansia* dominated module (MEcoral) were negatively related to fat percent (Figure 5C). MEwheat which contains less abundant OTUs from *Bifidobacterium* and *Ruminococcus*, was negatively associated with fat percent, and is positively associated with HbA_1c_C (Figure 5C). For the MUO individuals OTUs was clustered into four modules (Figure 4D). *Faecalibacterium* dominated module (MEgold) was negatively correlated with HbA_1c_ (Figure 5D). The MElightgreen module which contains Unclassified Ruminococcaceae, Clostridium XI, and *Bifidobacterium*, was positively associated with LDL-C, and was negatively correlated with VLDL-C, triglycerides, FPG and HbA_1c_ (Figure 5D). *Bacteroides* dominated module (MEwheat) was negatively associated with WC (Figure 5D).

## 4. Discussion

In this study, we sought to exploit microbiome profile of gut microbiota from a cohort of older adults, enabling a comprehensive comparison across metabolic health phenotypes among individuals with and without obesity. Our data indicate that metabolically healthy subjects without obesity had greater fecal microbiome diversity than their metabolically unhealthy (with MetS) counterparts. Specific microbes showed associations with metabolic health markers at different taxonomic level. Groups of co-occurrence microbes according to metabolic health phenotype provided new insights for microbiota–MetS–obesity relationships. Interestingly, no differences were noted for richness, diversity or taxonomy between the obese metabolic health phenotypes.

Emerging evidence indicates that the microbiome may play an important role in metabolic health, although mechanisms are still elusive and controversial [31]. Multiple studies have shown that alterations in the gut microbiota composition are linked to MetS in association studies [32,33,34]. Few studies report any relationship between gut microbiota diversity and MetS. We observed reduced alpha diversity of gut microbiota among older adults with MetS. However, this finding is only observed in subjects without obesity (MUNO subjects only), suggesting divergent influences of metabolic health and obesity status. As previously indicated, obesity is a heterogeneous condition and not all individuals with obesity display MetS features [4]. In addition, strong correlations between obesity and gut microbiota have been reported [19,35]. Thus, the influence of metabolic health status on gut microbiota may be more evident in the absence of obesity, whereas among individuals with obesity for whom many metabolic disturbances may exist, the influence of MetS status may be less evident. Regarding metabolic health classification, no gold standard metabolic health definition exists. Most studies focus on presence of MetS using a range of definitions or some of its components; others additionally include elevated C-reactive protein [36], thus giving rise to disparity in prevalence between studies. In contrast to our findings, a small study of Mexican women examining healthy (*n* = 25), obese (*n* = 17) and obese plus MetS (*n* = 25) subjects reported greater microbiota alpha diversity and richness among both groups of women with obesity relative to the healthy group [37]. Supporting those findings, a larger German study examining lean non-diabetic (*n* = 633) and obese individuals with and without T2DM (*n* = 153 and *n* = 494, respectively) revealed that obesity, but not T2DM, was associated with variation in gut microbiota [37]. Several studies have reported influences of specific bacterial taxa on metabolic health markers [32,38,39].

Our data show significant associations between gut microbiota and MetS, where a total of 12 unique taxa were significantly associated with 12 metabolic health markers. Decreased abundance of *Akkermansia, Anaerostipes* and *Phascolarctobacterium* was associated with elevated FPG levels, BMI and WC, and fat percent, respectively. These genera (*Akkermansia*, *Anaerostipes* and *Phascolarctobacterium)* have been identified as major producers of short chain fatty acids (SCFA) and are inversely related to fasting glucose level and obesity [40,41,42,43]. SCFAs are bioactive by-products of microbial fermentation of dietary fibers which have a range of metabolic functions relevant to MetS and obesity [37]. Butyrate, acetate and propionate are the three most abundant SCFAs in the human colon [44].

Our findings also demonstrate increased abundance of genus *Blautia* correlated with HDL-C concentrations. This is consistent with a previous study of 1914 Chinese individuals [39]. *Blautia* is identified as a butyric acid and acetic acid producer in the gut, which may have the potential to improve/reduce risk of MetS [45]. In addition, we found that *Clostridium* IV, *Collinsella* and *Anaerofilum* were positively correlated with serum lipids, such as total cholesterol, LDL-C, triglyceride and VLDL-C concentrations. Positive associations of anthropometric parameters such as BMI, fat percent and WC as well as blood pressure were observed with *Gemmiger*, *Parabacteroides*, *Mogibacterium* and *Allisonella*. Most of these genera have been reported to be pro-inflammatory bacteria and negatively related to metabolic health [39,46,47]. We have previously reported more favorable lipoprotein and inflammatory profiles among metabolically healthy adults regardless of obesity status, highlighting the importance of metabolic health for all.

The gut microbiota constitutes a complex ecosystem where abundant microbes perform specialized functions and cohesive interactions as a community. Moreover, complex relationships exist between gut microbiota and metabolic health markers. Therefore, integrative analysis of the microbiota and metabolome performed by co-occurrence may provide a better understanding of the functional variation of community and relationships with metabolic traits occurring in populations [32]. We determined functional modules of microbial communities based on co-occurrence according to the metabolic health phenotype. Furthermore, these groups were used to identify associations with traits. The different functional groups determined in MHNO, MUNO, MHO and MUO confirmed the heterogeneous condition of the metabolic health obese and non-obese phenotypes. However, OTU co-occurrence analysis also confirmed generality of the gut microbiota. For instance, the butyrate-producing bacteria *Faecalibacterium* dominated group exhibited negative correlations with metabolic health markers including fat percent, triglycerides and HbA_1c_. Moreover, the *Akkermansia* dominated group was negatively associated with fasting glucose and this genus also showed an inverse association with fasting glucose, confirming the consistency between different analyses. In addition, OTU co-occurrence-based associations with metabolic health markers also revealed some new insights into the relationships between community and traits that were not observed in analysis of single taxonomy-based associations. For example, the *Bifidobacterium* dominated group, whose genus was not significantly associated with metabolic health markers in linear regression analysis, was negatively correlated with VLDL-C, triglycerides, FPG and HbA_1c_. This is consistent with a previous study which found that *Bifidobacterium* potentially prevented high fat diet-induced diabetes in mice [48].

The current investigation has several strengths including a cohort of well characterized older adults with evaluable data, permitting a large range of potential confounders to be included in the analyses, and equal sex representation (50.8% male) who are representative of the source population reported in the national census data [25]. Limitations of our study should also be considered. Due to the cross-sectional nature of the study, causation cannot be determined. We explored many potential associations, increasing the risk of detecting false positives/type 1 errors. To correct for the multiple testing performed *p* values were corrected with the Benjamini-Hochberg false-discovery rate in the multivariate analyses. The findings regarding alpha diversity and co-occurrence analysis were not in agreement with each other in the obese and non-obese individuals, suggesting limited power to detect relationships and variations in the complex metabolic intrinsic connections. We did not examine bowel function or stool consistency, which has recently been linked with microbiome richness [49]. It is also worth considering obesity classification. BMI does not discriminate between lean and fat mass. It has been demonstrated that 50% of individuals with obesity may be considered to be metabolically healthy when classified using dual energy X-ray absorptiometry-derived body fat (the gold standard measurement) compared to a third by BMI [50]. Interestingly, we have previously demonstrated that in individuals with MetS, using both fat percentage and BMI to classify obesity may be superior in identifying cardiometabolic risk factors than BMI alone [51]. Finally, previous population-based studies had a larger age distribution, whereas our cohort, by virtue of its starting date and recall nature, involves only older Irish people, which may limit the generalizability of our findings. More population-based cohort studies are needed to confirm whether there are universal biomarkers of fecal sample-derived microbiota from subjects with MetS, with and without obesity.

## 5. Conclusions

In conclusion, our results suggest that specific metabolic health markers and metabolic heath status are associated with certain gut microbiota whose functions provide biologically plausible explanations underlying these relationships and metabolic alterations. We report greater microbiome diversity among the metabolically healthy non-obese individuals relative to their metabolically unhealthy counterparts. Certain microbes showed associations with metabolic health markers at different taxonomic levels. Co-occurrence network analysis revealed different patterns among metabolic groups and individual metabolic health marker correlations with groups of microbes. Our data provide a significant biological resource to confirm previously reported associations between gut microbiota and MetS and support further studies. The previously reported reduced diversity observed in obesity [12,52] may have hindered detection of differences between the obese metabolic health phenotypes. Functional and mechanistic studies are warranted to clarify the interactions between gut microbiota and specific metabolic phenotypes, to disentangle the specific microbial profiles unique to obesity and metabolic health status and identify unifying mechanisms. More evidence is required to identify biomarkers for MetS.

## Figures and Tables

**Table 1:** Descriptive characteristics of the study population according to metabolic health phenotype.

Characteristics	All	FDR	MHNO	MUNO	FDR	MHO	MUO	FDR
	(*n* = 382)		(*n* = 191)	(*n* = 61)		(*n* = 66)	(*n* = 64)	
Age (years) ^a^	69.91 (5)	0.174	69 (6)	70 (5)	0.387	69 (5)	68.5 (4)	0.442
Sex (male) ^b^	194 (50.79)	0.425	93 (48.69)	36 (59.01)	0.757	27 (40.91)	38 (59.37)	0.822
BMI (kg/m^2^) ^a^	28.82 (5.52)	**<0.001**	26.11 (3.66)	27.76 (2.39)	**<0.001**	32.44 (3.57)	34.32 (4.91)	**0.042**
WC (cm) ^c^	102.1 (13.36)	**<0.001**	95.1 (10.12)	101.2 (8.62)	**<0.001**	109.4 (9.49)	117.12 (10.31)	**0.002**
Fat percent (%) ^a^	30.49 (9.87)	**<0.001**	27.7(6.97)	32.6(6.08)	**0.005**	30.7(6.25)	34.15(6.24)	0.198
SBP (mmHg) ^a^	129.16 (25.38)	**<0.001**	123.5 (22.5)	139.5 (20.5)	**<0.001**	123.75 (23.5)	135 (26.62)	**0.006**
DBP (mmHg) ^a^	73.8 (13)	**0.003**	71 (12.5)	77 (10.5)	**<0.001**	74 (12.5)	74.75 (14)	0.994
HbA_1c_ (mmol/mol) ^a^	40.34 (4)	**<0.001**	38 (4)	40 (7)	**<0.001**	39 (4)	40 (7)	**0.006**
FPG (mmol/L) ^a^	5.48 (0.8)	**<0.001**	4.9 (0.6)	5.8 (1.7)	**<0.001**	5.05 (0.4)	5.9 (1.15)	**<0.001**
Total cholesterol (mmol/L) ^a^	5 (1.3)	0.065	5 (1.2)	5 (2)	0.831	5 (1.1)	4.6 (1.13)	0.082
HDL-C (mmol/L) ^a^	1.42 (0.47)	**<0.001**	1.47 (0.5)	1.18 (0.39)	**<0.001**	1.37 (0.27)	1.14 (0.28)	**<0.001**
LDL-C (mmol/L) ^a^	2.98 (1.1)	**0.002**	3 (1.2)	2.9 (1.5)	0.948	2.9 (1)	2.5 (1.02)	**0.005**
VLDL-C (mg/dL) ^a^	0.59 (0.3)	**<0.001**	0.5 (0.1)	0.7 (0.4)	**<0.001**	0.5 (0.17)	0.8 (0.6)	**<0.001**
Triglycerides (mmol/L) ^a^	1.3 (0.66)	**<0.001**	0.95 (0.45)	1.52 (0.87)	**<0.001**	1.14 (0.35)	1.78 (1.11)	**<0.001**
Current smoker ^b^	24 (6.28)	0.641	10 (5.23)	3 (4.92)	0.757	5 (7.57)	6 (9.37)	0.644
Current alcohol consumers ^b^	248 (64.92)	0.98	126 (65.96)	42 (68.85)	0.892	39 (59.09)	41 (64.06)	1
T2DM ^b^	32 (8.38)	**<0.001**	7 (3.66)	3 (4.91)	**<0.001**	12 (18.18)	10 (15.63)	0.122
CVD ^b^	299 (78.27)	**0.012**	139 (72.77)	49 (80.33)	**0.043**	54 (81.82)	57 (89.06)	0.119
On cholesterol lowering medication ^b^	203 (53.14)	**0.012**	92 (48.16)	29 (47.54)	0.133	38 (57.58)	44 (68.75)	**0.026**
On BP lowering medication ^b^	257 (67.28)	**<0.001**	100 (52.36)	40 (80.33)	**<0.001**	59 (89.39)	58 (90.63)	**0.001**
**Dietary information**								
DASH score ^a^	24 (5.7)	**0.032**	25 (7)	23 (7)	0.098	24 (6.75)	22 (8)	0.198
Energy (kcal per day) ^a^	1 951 (998)	0.566	1 693 (834)	1 873 (1 422)	0.249	2 042 (1 131)	1 808 (864)	0.492
Protein (%kcal) ^a^	18.96 (5.38)	0.542	18.11 (5.53)	18.4 (4.93)	0.639	18.33 (4.85)	19.03 (5.91)	0.485
Carbohydrate (%kcal) ^a^	48.3 (9.96)	**0.004**	49.47 (8.24)	48.36 (9.96)	0.187	48.78 (11.26)	45.22 (11.42)	0.082
Fiber (%kcal) ^a^	2.4 (0.81)	**0.037**	2.38 (0.81)	2.29 (0.84)	0.087	2.2 (0.88)	2.23 (0.68)	0.41
Fat (%kcal) ^a^	34.34 (8.4)	**0.011**	33.66 (7.92)	35.33 (10.71)	0.324	35.24 (8.81)	36.88 (6.06)	0.062
Monounsaturated fat (%kcal) ^a^	11.13 (3.19)	**0.005**	10.77 (3.11)	11.09 (3.89)	0.204	11.54 (4.02)	11.61 (2.51)	0.41
Polyunsaturated fat (%kcal) ^a^	5.8 (2.43)	0.1	5.38 (2.31)	5.38 (2.24)	0.937	5.77 (2.02)	6.08 (2.87)	0.357
Saturated fat (%kcal) ^a^	12.32 (4.8)	**0.041**	11.42 (4.16)	12.62 (5.37)	0.187	11.93 (3.24)	12.6 (4.53)	0.163

FDR < 0.05 are in bold. ^a^ Non-Normally distributed numeric variables were displayed as median (interquartile range). ^b^ Category variables and displayed as count (%). ^c^ Normally distributed variables were displayed as mean (SD).

## Supplementary Materials

The following are available online at https://www.mdpi.com/2072-6643/12/8/2364/s1. Figure S1. Principal co-ordinate analysis between MUNO and MHNO groups based on different dissimilarity distance matrix. Figure S2. Principal co-ordinate analysis between MUO and MHO groups based on different dissimilarity distance matrix. Figure S3. Gut microbiota composition of MCR cohort at the phylum level. Figure S4. Gut microbiota composition of MCR cohort at the family level. Figure S5. Gut microbiota composition of MCR cohort at the genus level. Table S1. Linear regression between MetS risk factors and alpha diversity. Table S2. Permutational multivariate analysis of variance using Bray-Curtis distance matrices. Table S3. Abundant taxa at phylum level within metabolic health groups. Table S4. Abundant taxa at family level within metabolic health groups. Table S5. Abundant taxa at genus level within metabolic health groups. Table S6. Significant results of linear regression between metabolic health markers and phylum relative abundance. Table S7. Significant results of linear regression between metabolic health markers and family relative abundance. Table S8. Significant results of linear regression between metabolic health markers and genus relative abundance.
