# Supplementary material for: Gut Microbiota Associations with Metabolic Health and Obesity Status in Older Adults
Source: Nutrients. 2020 Aug 7;12(8):2364. doi: 10.3390/nu12082364 (PMC7468966; doi:10.3390/nu12082364)
Supplement: Supplementary file 1 [file nutrients-12-02364-s001.pdf]

Title: Gut microbiota composition and diversity and its relation to metabolic health and obesity status in older adults

Authors: Xiaozhong Zhong[1, 2], Janas M. Harrington[3], Seán R. Millar[3], Ivan J. Perry[3], Paul W. O'Toole[2], Catherine M. Phillips[3, 4]

Affiliations:

[1]National Engineering Laboratory for Cereal Fermentation Technology, Jiangnan University, Wuxi 214122, PR China

[2]School of Microbiology and APC Microbiome Ireland, University College Cork, Cork, Ireland

[3]HRB Centre for Health and Diet Research, School of Public Health, University College Cork, Cork, Ireland

[4]School of Public Health, Physiotherapy, and Sports Science, University College Dublin, Dublin 4, Ireland

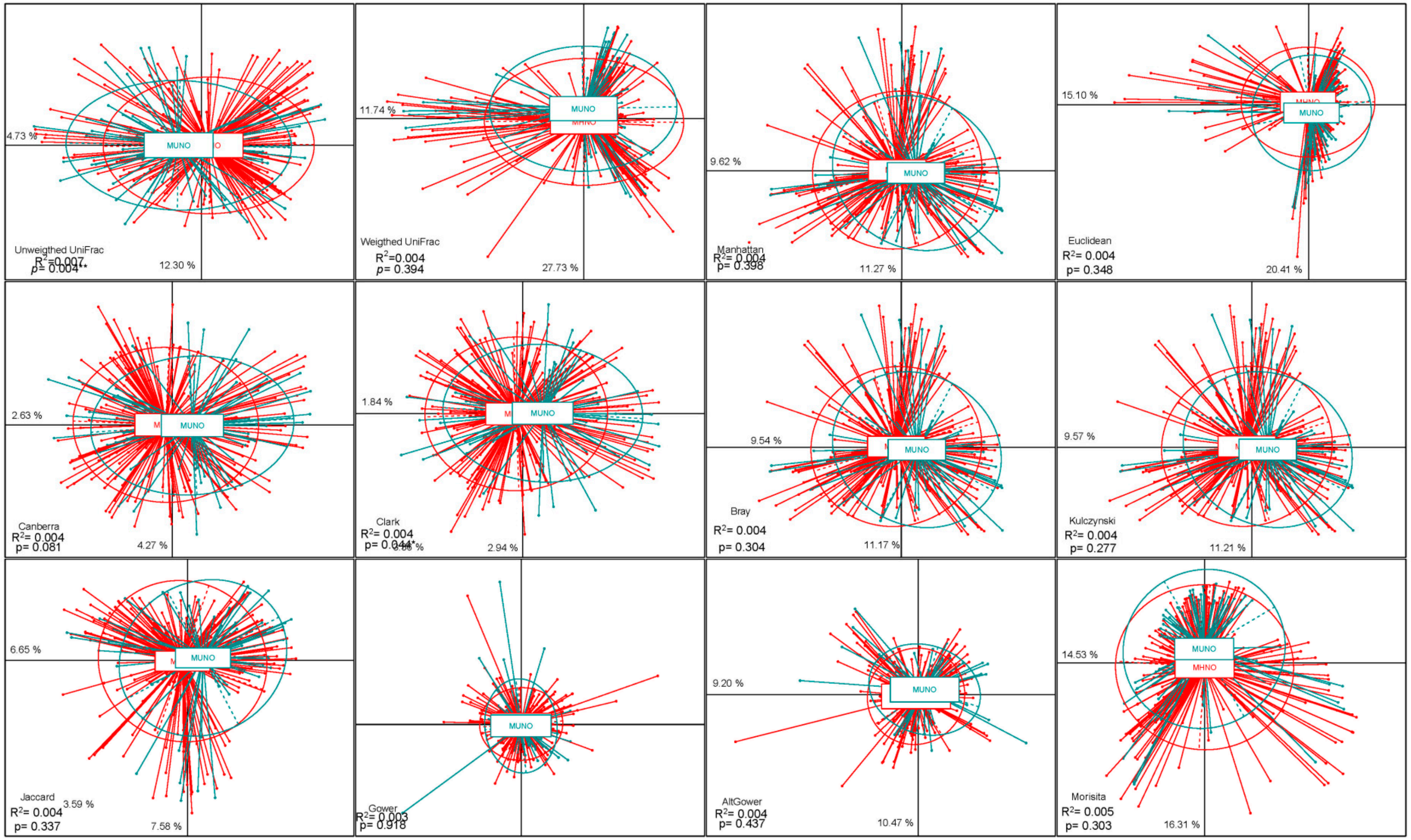

Fig. S1 Principal co-ordinate analysis between MUNO and MHNO groups based on different dissimilarity distance matrix. Subjects are colour coded according to Mets group; MHNO (red), MUNO (green). P-values were calculated from *adonis* test, *p < 0.05.

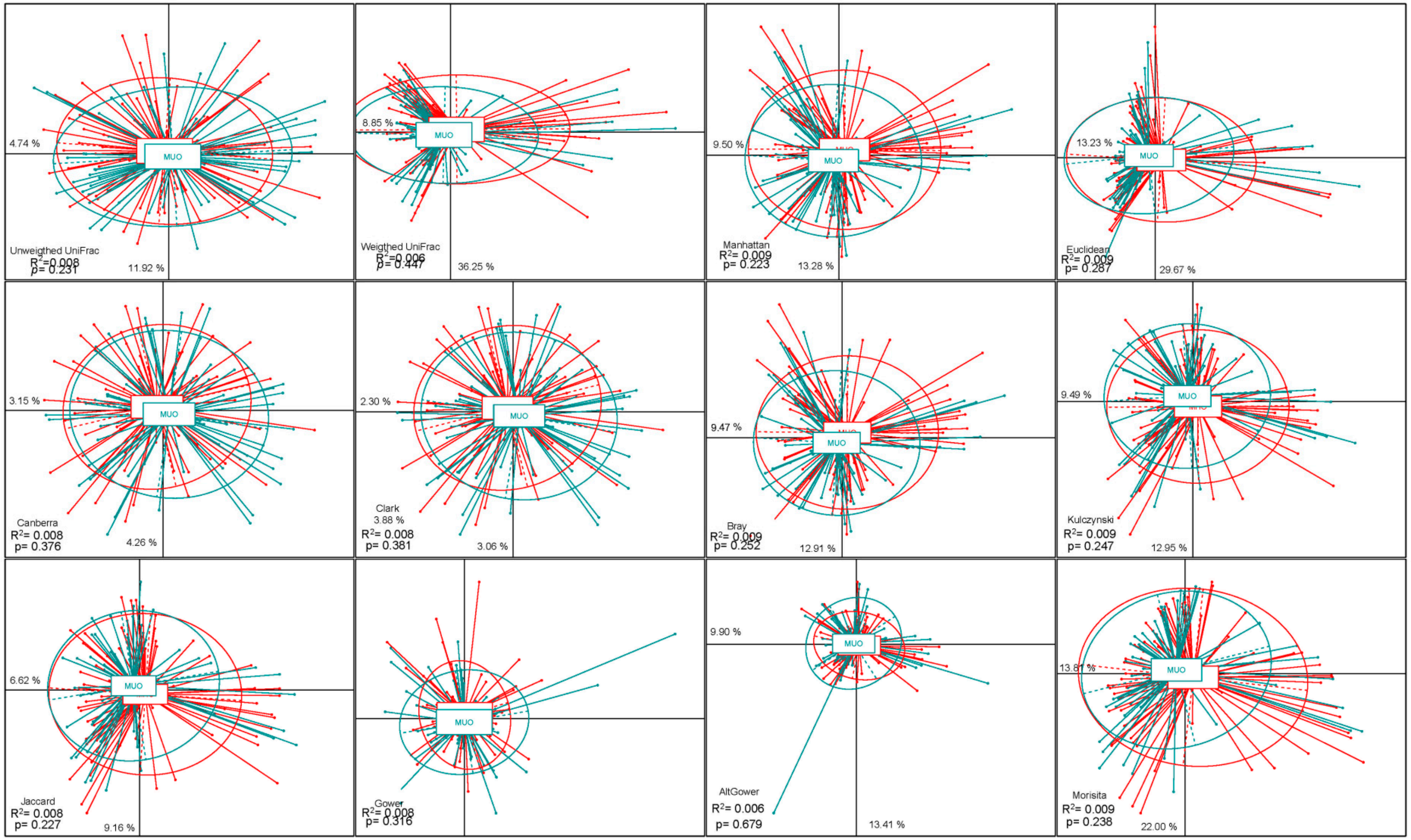

Fig. S2 Principal co-ordinate analysis between MUO and MHO groups based on different dissimilarity distance matrix. Subjects are colour coded according to Mets group; MHO (red), MUO (green). P-values were calculated from *adonis* test, $^*p < 0.05$

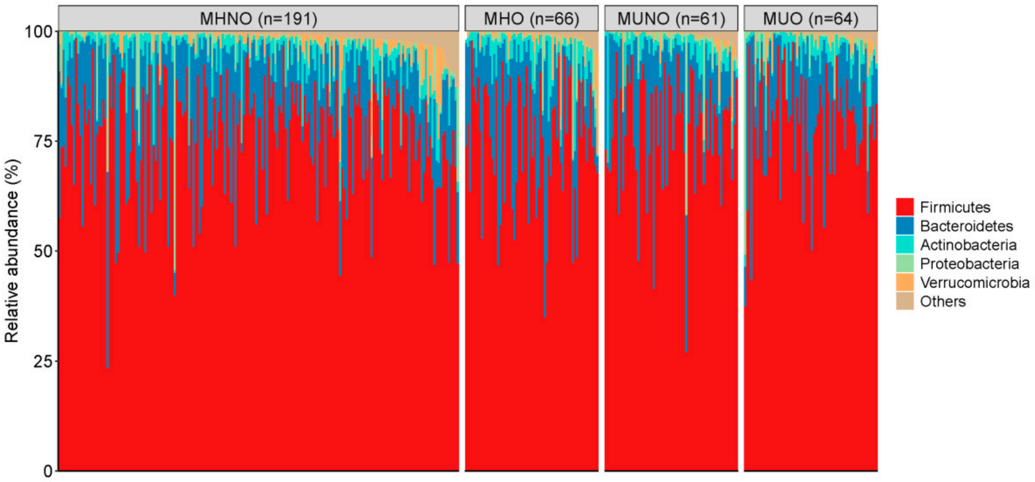

Fig. S3 Gut microbiota composition of MCR cohort at the phylum level.

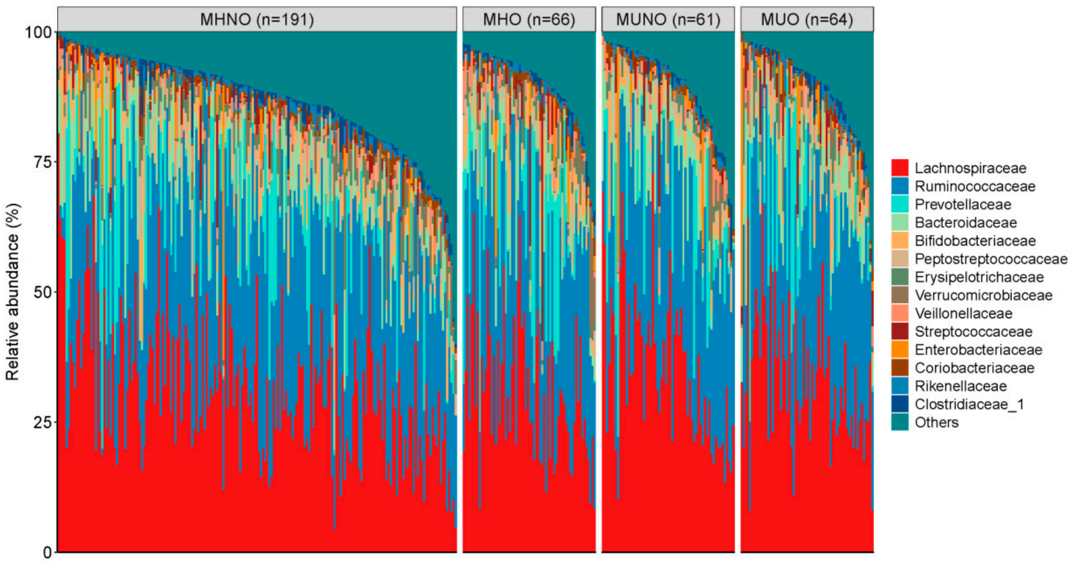

Fig. S4 Gut microbiota composition of MCR cohort at the family level.

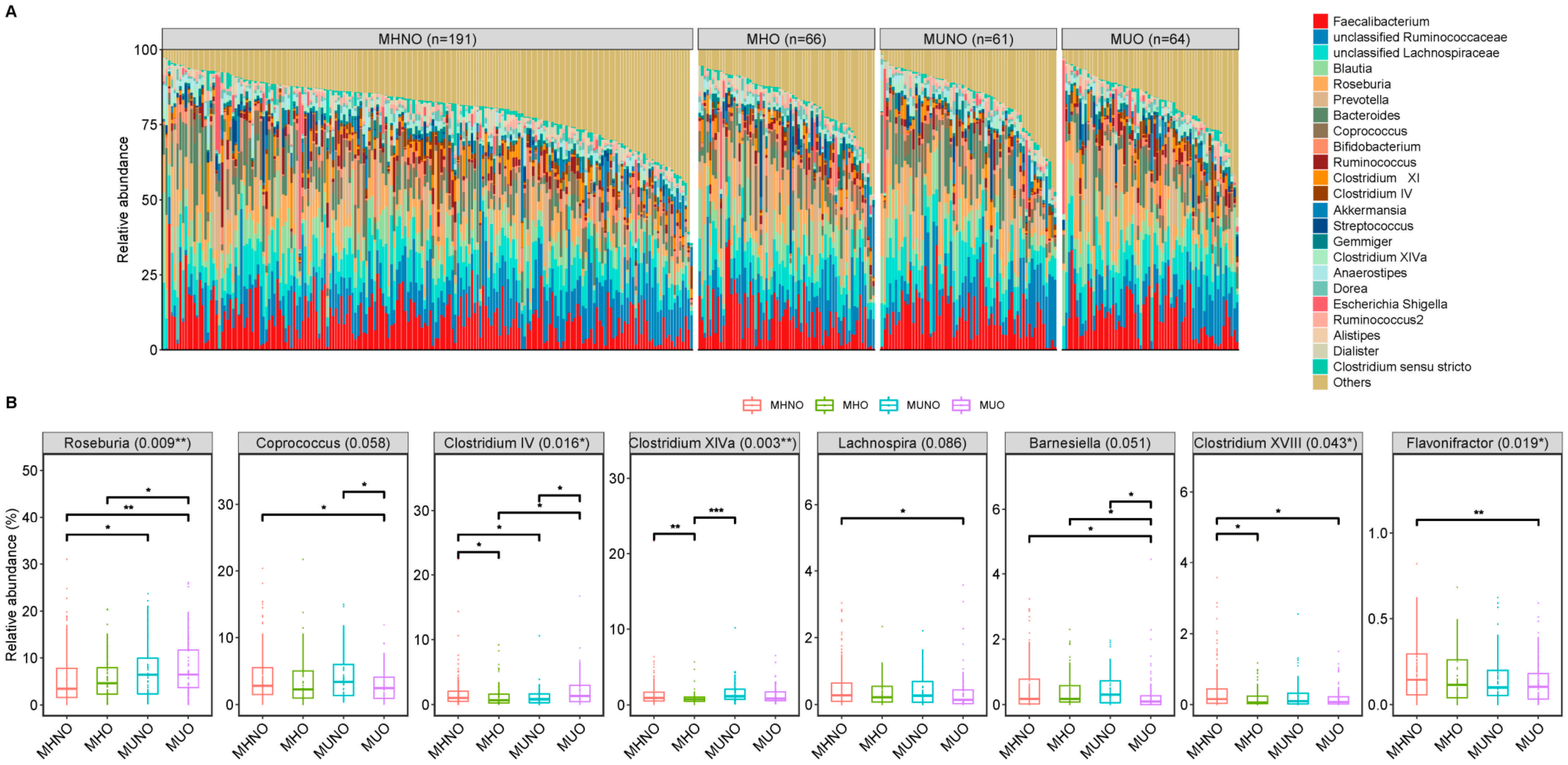

Fig. S5 Gut microbiota composition of MCR cohort at the genus level. (A) bar chart, (B) significantly different genus between MetS groups

Table S1 Linear regression between MetS risk factors and alpha diversity

Variables	Chao1				PD whole tree				Simpson				Shannon				Observed species			
	Coefficient	R^2	p value	FDR	Coefficient	R^2	p value	FDR	Coefficient	R^2	p value	FDR	Coefficient	R^2	p value	FDR	Coefficient	R^2	p value	FDR
**BMI**	-1.167	0.033	0.355	0.476	-0.105	0.045	0.210	0.359	-0.001	0.048	0.071	0.171	-0.012	0.053	0.095	0.228	-1.307	0.045	0.165	0.330
**SBP**	-0.437	0.033	0.239	0.410	-0.035	0.045	0.155	0.311	0.000	0.040	0.614	0.670	-0.002	0.047	0.452	0.602	-0.278	0.042	0.317	0.476
**DBP**	-0.580	0.032	0.357	0.476	-0.046	0.044	0.266	0.389	0.000	0.041	0.430	0.574	0.002	0.047	0.610	0.731	-0.309	0.041	0.510	0.557
**WC**	-0.608	0.034	0.196	0.393	-0.033	0.043	0.292	0.389	0.000	0.050	**0.041**	0.124	-0.005	0.054	0.072	0.216	-0.458	0.044	0.193	0.331
**HbA1C**	-0.549	0.032	0.554	0.665	-0.046	0.044	0.456	0.515	-0.001	0.045	0.125	0.251	-0.008	0.053	0.136	0.234	-0.522	0.043	0.451	0.557
**Fasting glucose**	-9.217	0.039	0.084	0.202	-0.654	0.050	0.065	0.157	-0.005	0.051	**0.026**	0.105	-0.064	0.057	**0.037**	0.147	-7.110	0.050	0.074	0.178
**Total cholesterol**	14.802	0.048	**0.011**	**0.034**	1.063	0.061	**0.006**	**0.037**	0.006	0.057	**0.013**	0.077	0.089	0.065	**0.008**	**0.045**	11.850	0.061	**0.007**	**0.040**
**Triglyceride**	-24.634	0.050	**0.006**	**0.022**	-1.346	0.054	**0.023**	0.069	-0.003	0.042	0.399	0.574	-0.069	0.051	0.176	0.265	-14.507	0.053	**0.029**	0.088
**HDL-C**	2.815	0.031	0.859	0.859	-0.193	0.041	0.854	0.854	0.003	0.041	0.587	0.670	0.038	0.047	0.673	0.734	1.948	0.041	0.869	0.869
**Fat percent**	-0.443	0.031	0.649	0.708	-0.046	0.042	0.472	0.515	0.000	0.040	0.840	0.840	-0.001	0.046	0.920	0.920	-0.477	0.042	0.511	0.557
**LDL-C**	22.537	0.061	**0.001**	**0.006**	1.571	0.074	**0.000**	**0.003**	0.007	0.057	**0.010**	0.077	0.110	0.068	**0.003**	**0.038**	16.454	0.069	**0.001**	**0.008**
**VLDL-C**	-56.416	0.052	**0.004**	**0.022**	-2.968	0.054	**0.023**	0.069	-0.008	0.042	0.306	0.524	-0.172	0.052	0.126	0.234	-32.851	0.053	**0.025**	0.088

P values < 0.05 are in bold.

Permutational multivariate analysis adjusted for age, sex, disease status (CVD and T2DM), medication use (blood pressure, cholesterol lowering and diabetes medication), lifestyle factors (DASH score, smoking status and alcohol consumption)

Table S2 Permutational multivariate analysis of variance using Bray-Curtis distance matrices

Variable	R^2	p value	FDR
**Glucose**	0.004	**0.016**	0.088
**BMI**	0.004	**0.022**	0.088
**HbA1c**	0.004	**0.022**	0.088
**WC**	0.004	**0.048**	0.144
**Fat percent**	0.004	0.067	0.161
**LDL-C**	0.003	0.138	0.249
**Triglyceride**	0.003	0.145	0.249
**VLDL-C**	0.003	0.226	0.339
**HDL-C**	0.003	0.439	0.533
**SBP**	0.003	0.444	0.533
**Total Cholesterol**	0.002	0.520	0.567
**DBP**	0.002	0.579	0.579

P value < 0.05 are in bold.

Permutational multivariate analysis adjusted for age, sex, disease status (CVD and T2DM), medication use (blood pressure, cholesterol lowering and diabetes medication), lifestyle factors (DASH score, smoking status and alcohol consumption).

Table S3 Abundant taxa at phylum level within metabolic health groups.

Subjects Without Obesity					
**Phylum**	**MHNO (%)**	**MUNO (%)**	**Log2FC**	**P value**	**Adjusted p value**
**Euryarchaeota**	0.067 ± 0.12	0.051 ± 0.091	-1.364	**0.023**	0.09
**Bacteroidetes**	15.561 ± 11.806	15.377 ± 12.041	0.214	0.254	0.677
**Actinobacteria**	3.569 ± 4.275	2.933 ± 4.057	-0.205	0.445	0.889
**Verrucomicrobia**	1.831 ± 3.822	1.306 ± 3.641	-1.094	0.555	0.889
**Lentisphaerae**	0.012 ± 0.036	0.017 ± 0.079	-0.211	0.794	0.911
**Firmicutes**	75.944 ± 13.024	77.489 ± 13.516	0.022	0.86	0.911
**Proteobacteria**	1.922 ± 5.445	2.176 ± 6.093	0.095	0.911	0.911
**Subjects With Obesity**					
**Phylum**	**MHO (%)**	**MUO (%)**	**Log2FC**	**P value**	**Adjusted p value**
**Firmicutes**	75.041 ± 14.418	78.358 ± 12.944	0.295	**0.007**	0.054
**Actinobacteria**	4.007 ± 4.409	3.185 ± 3.756	-0.429	0.067	0.178
**Bacteroidetes**	16.69 ± 14.585	14.271 ± 12.41	-0.088	0.761	0.968
**Verrucomicrobia**	1.584 ± 3.964	1.234 ± 2.455	-0.557	0.824	0.968
**Euryarchaeota**	0.071 ± 0.168	0.072 ± 0.193	0.193	0.893	0.968
**Lentisphaerae**	0.015 ± 0.074	0.005 ± 0.023	-0.144	0.929	0.968
**Proteobacteria**	1.88 ± 4.268	2.303 ± 6.731	-0.017	0.968	0.968

P value < 0.05 are in bold.

Adjusted p values were calculated by DESeq2 test with Benjamini–Hochberg correction.

Permutational multivariate analysis adjusted for age, sex, disease status (CVD and T2DM), medication use (blood pressure, cholesterol lowering and diabetes medication), lifestyle factors (DASH score, smoking status and alcohol consumption).

Table S4 Abundant taxa at family level within metabolic health groups.

Subjects Without Obesity					
	**MHNO (%)**	**MUNO (%)**	**Log2FC**	**P value**	**Adjusted p value**
**Lachnospiraceae**	31.40 ± 13.63	35.20 ± 14.57	0.436	**0.003**	**0.04**
**Bacteroidaceae**	5.70 ± 6.789	6.74 ± 7.68	0.732	**0.013**	0.123
**Methanobacteriaceae**	0.06 ± 0.12	0.05 ± 0.09	-1.174	**0.041**	0.283
**Pasteurellaceae**	0.06 ± 0.29	0.06 ± 0.25	1.352	**0.049**	0.283
**Ruminococcaceae**	28.31 ± 9.95	28.95 ± 10.28	0.197	0.101	0.447
**Micrococcaceae**	0.005 ± 0.01	0.007 ± 0.02	0.919	0.108	0.447
**Streptococcaceae**	1.15 ± 2.75	1.24 ± 3.45	0.591	0.176	0.609
**Actinomycetaceae**	0.02 ± 0.03	0.02 ± 0.06	0.439	0.213	0.609
**Oxalobacteraceae**	0.004 ± 0.01	0.006 ± 0.01	0.67	0.228	0.609
**Porphyromonadaceae**	1.04 ± 1.05	0.97 ± 0.89	0.315	0.231	0.609
**Coriobacteriaceae**	1.12 ± 1.18	0.95 ± 0.78	-0.194	0.276	0.64
**Erysipelotrichaceae**	1.70 ± 1.97	1.51 ± 1.92	0.225	0.287	0.64
**Peptostreptococcaceae**	2.20 ± 2.82	1.65 ± 2.01	-0.307	0.366	0.755
**Peptococcaceae_1**	0.03 ± 0.07	0.03 ± 0.06	-0.586	0.39	0.755
**Rikenellaceae**	1.13 ± 1.61	0.82 ± 0.78	0.144	0.606	0.971
**Sutterellaceae**	0.13 ± 0.17	0.11 ± 0.20	-0.162	0.673	0.971
**Acidaminococcaceae**	0.63 ± 0.82	0.63 ± 0.94	-0.179	0.684	0.971
**Verrucomicrobiaceae**	1.79 ± 3.80	1.29 ± 3.62	-0.75	0.718	0.971
**Bifidobacteriaceae**	2.42 ± 3.71	1.95 ± 3.60	-0.117	0.737	0.971
**Lactobacillaceae**	0.50 ± 2.63	0.56 ± 2.17	0.884	0.751	0.971
**Clostridiaceae_1**	1.17 ± 2.02	0.81 ± 1.44	-0.288	0.771	0.971
**Victivallaceae**	0.01 ± 0.03	0.016 ± 0.07	-0.18	0.825	0.971
**Eubacteriaceae**	0.01 ± 0.02	0.004 ± 0.008	-0.12	0.854	0.971
**Clostridiales_Incertae_Sedis_XIII**	0.03 ± 0.04	0.032 ± 0.06	-0.089	0.876	0.971
**Prevotellaceae**	6.51 ± 11.41	5.904 ± 11.19	-0.197	0.945	0.971
**Veillonellaceae**	1.25 ± 1.91	1.471 ± 2.10	-0.084	0.95	0.971
**Enterobacteriaceae**	1.15 ± 5.15	1.375 ± 5.98	-0.137	0.965	0.971
**Desulfovibrionaceae**	0.19 ± 0.26	0.25 ± 0.34	0.012	0.971	0.971
**Subjects With Obesity**					
	**MHO (%)**	**MUO (%)**	**Log2FC**	**P value**	**Adjusted p value**
**Ruminococcaceae**	27.98 ± 9.09	29.23 ± 9.24	0.26	0.051	0.781
**Bifidobacteriaceae**	2.65 ± 3.70	2.03 ± 3.41	-0.709	0.061	0.781
**Lachnospiraceae**	30.83 ± 13.40	33.37 ± 12.74	0.241	0.081	0.781
**Desulfovibrionaceae**	0.18 ± 0.26	0.19 ± 0.25	0.57	0.138	0.921
**Coriobacteriaceae**	1.33 ± 1.42	1.12 ± 1.29	-0.281	0.159	0.921
**Peptostreptococcaceae**	1.95 ± 2.26	1.98 ± 2.61	0.459	0.202	0.968
**Bacteroidaceae**	4.83 ± 5.45	5.56 ± 6.40	0.346	0.276	0.968
**Rikenellaceae**	1.23 ± 1.68	1.04 ± 1.34	-0.348	0.332	0.968
**Erysipelotrichaceae**	2.08 ± 2.30	1.84 ± 2.67	-0.222	0.425	0.968
**Clostridiaceae_1**	0.92 ± 1.29	0.95 ± 1.45	0.321	0.452	0.968
**Clostridiales_Incertae_Sedis_XIII**	0.05 ± 0.07	0.04 ± 0.07	-0.407	0.468	0.968

**Peptococcaceae_1**	0.03 ± 0.06	0.03 ± 0.09	-0.474	0.591	0.968
**Micrococcaceae**	0.004 ± 0.01	0.01 ± 0.03	0.364	0.592	0.968
**Actinomycetaceae**	0.02 ± 0.03	0.02 ± 0.03	0.197	0.629	0.968
**Porphyromonadaceae**	0.90 ± 0.77	0.72 ± 0.90	-0.152	0.652	0.968
**Pasteurellaceae**	0.02 ± 0.05	0.06 ± 0.25	0.596	0.684	0.968
**Oxalobacteraceae**	0.004 ± 0.009	0.004 ± 0.01	0.359	0.689	0.968
**Methanobacteriaceae**	0.07 ± 0.16	0.07 ± 0.19	-0.282	0.696	0.968
**Eubacteriaceae**	0.009 ± 0.04	0.007 ± 0.03	-0.331	0.709	0.968
**Prevotellaceae**	9.19 ± 14.74	6.49 ± 12.15	-0.846	0.794	0.968
**Verrucomicrobiaceae**	1.58 ± 3.96	1.23 ± 2.45	-0.578	0.797	0.968
**Enterobacteriaceae**	1.23 ± 4.18	1.78 ± 6.74	0.654	0.838	0.968
**Streptococcaceae**	2.19 ± 6.74	1.69 ± 2.99	0.252	0.876	0.968
**Sutterellaceae**	0.23 ± 0.43	0.12 ± 0.15	-0.134	0.884	0.968
**Victivallaceae**	0.02 ± 0.07	0.005 ± 0.02	-0.134	0.933	0.968
**Veillonellaceae**	2.08 ± 2.81	1.44 ± 2.68	0.113	0.951	0.968
**Lactobacillaceae**	0.26 ± 0.81	1.70 ± 6.10	0.199	0.951	0.968
**Acidaminococcaceae**	0.48 ± 0.66	0.48 ± 0.54	-0.018	0.968	0.968

P values < 0.05 are in bold.

Adjusted p values were calculated by DESeq2 test with Benjamini–Hochberg correction.

Permutational multivariate analysis adjusted for age, sex, disease status (CVD and T2DM), medication use (blood pressure, cholesterol lowering and diabetes medication), lifestyle factors (DASH score, smoking status and alcohol consumption).

Table S5 Abundant taxa at genus level within metabolic health groups.

Subjects Without Obesity					
**Genus**	**MHNO (%)**	**MUNO (%)**	**Log2FC**	**P value**	**Adjusted p value**
**Flavonifractor**	0.16 ± 0.15	0.12 ± 0.16	-0.628	**0.037**	0.89
**Roseburia**	5.60 ± 5.50	7.45 ± 6.45	0.354	0.093	0.89
**Clostridium_XI**	2.19 ± 2.81	1.64 ± 2.00	-0.597	0.096	0.89
**Rothia**	0.005 ± 0.01	0.007 ± 0.01	0.912	0.102	0.89
**Haemophilus**	0.06 ± 0.28	0.06 ± 0.24	1.142	0.105	0.89
**unclassified Lachnospiraceae**	9.34 ± 4.26	10.62 ± 4.47	0.123	0.114	0.89
**Butyricicoccus**	0.21 ± 0.19	0.25 ± 0.20	0.322	0.14	0.89
**Dorea**	1.29 ± 0.94	1.48 ± 1.24	0.227	0.144	0.89
**unclassified Oxalobacteraceae**	0.004 ± 0.01	0.006 ± 0.01	0.841	0.153	0.89
**unclassified Coriobacteriaceae**	0.24 ± 0.31	0.19 ± 0.24	-0.419	0.17	0.89
**unclassified Peptostreptococcaceae**	0.007 ± 0.01	0.004 ± 0.01	-1.115	0.17	0.89
**Slackia**	0.041 ± 0.09	0.04 ± 0.07	0.75	0.173	0.89
**unclassified Ruminococcaceae**	12.37 ± 7.62	11.64 ± 8.62	-0.278	0.192	0.89
**Holdemania**	0.012 ± 0.02	0.012 ± 0.01	-0.506	0.195	0.89
**Bifidobacterium**	2.42 ± 3.70	1.95 ± 3.60	-0.486	0.198	0.89
**Faecalibacterium**	10.78 ± 7.32	12.45 ± 8.35	0.265	0.198	0.89
**Barnesiella**	0.48 ± 0.67	0.48 ± 0.52	0.495	0.228	0.945
**Lachnospira**	0.86 ± 0.93	0.69 ± 0.66	-0.318	0.236	0.945
**Gemmiger**	1.31 ± 1.19	1.46 ± 1.58	0.279	0.273	0.992
**Ruminococcus**	2.36 ± 2.83	1.96 ± 2.05	-0.291	0.303	0.992
**Butyricimonas**	0.05 ± 0.07	0.04 ± 0.05	-0.431	0.318	0.992
**Allisonella**	0.02 ± 0.11	0.03 ± 0.05	0.579	0.354	0.992
**Methanobrevibacter**	0.06 ± 0.11	0.05 ± 0.08	-1.047	0.363	0.992
**Sporobacter**	0.004 ± 0.0	0.003 ± 0.01	-0.46	0.371	0.992
**Clostridium_IV**	0.78 ± 1.01	0.78 ± 1.35	0.222	0.417	0.992
**Ruminococcus2**	0.90 ± 0.92	1.06 ± 1.28	-0.161	0.418	0.992
**Sutterella**	0.09 ± 0.15	0.07 ± 0.19	-0.41	0.438	0.992
**Peptococcus**	0.03 ± 0.07	0.03 ± 0.06	-0.51	0.473	0.992
**Mogibacterium**	0.03 ± 0.05	0.03 ± 0.06	-0.437	0.484	0.992
**Collinsella**	0.67 ± 0.83	0.61 ± 0.67	-0.209	0.502	0.992
**Clostridium_XVIII**	0.36 ± 0.56	0.26 ± 0.4	-0.226	0.51	0.992
**Asaccharobacter**	0.04 ± 0.06	0.03 ± 0.05	-0.242	0.534	0.992
**unclassified Desulfovibrionaceae**	0.03 ± 0.10	0.02 ± 0.07	-0.496	0.544	0.992
**Parasutterella**	0.04 ± 0.10	0.04 ± 0.07	0.369	0.548	0.992
**Howardella**	0.04 ± 0.07	0.04 ± 0.05	-0.28	0.555	0.992
**Alistipes**	1.13 ± 1.62	0.82 ± 0.78	-0.17	0.573	0.992
**Anaerofilum**	0.003 ± 0.01	0.005 ± 0.009	0.281	0.589	0.992
**Turicibacter**	0.16 ± 0.35	0.11 ± 0.22	-0.297	0.597	0.992
**Anaerotruncus**	0.008 ± 0.01	0.01 ± 0.023	0.201	0.604	0.992
**Victivallis**	0.01 ± 0.03	0.03 ± 0.07	0.433	0.629	0.992
**Eggerthella**	0.02 ± 0.05	0.01 ± 0.02	-0.244	0.637	0.992

**Clostridium_XlVb**	0.15 ± 0.27	0.11 ± 0.19	-0.171	0.641	0.992
**Coprococcus**	3.93 ± 3.54	3.92 ± 3.26	0.086	0.688	0.992
**Clostridium_sensu_stricto**	1.16 ± 2.01	0.78 ± 1.42	-0.402	0.702	0.992
**Actinomyces**	0.02 ± 0.026	0.02 ± 0.05	0.127	0.723	0.992
**unclassified Erysipelotrichaceae**	0.68 ± 1.16	0.49 ± 0.99	-0.151	0.727	0.992
**Parabacteroides**	0.16 ± 0.30	0.20 ± 0.29	0.148	0.728	0.992
**Enterorhabdus**	0.03 ± 0.04	0.02 ± 0.04	0.222	0.736	0.992
**Akkermansia**	1.79 ± 3.80	1.29 ± 3.62	-0.863	0.738	0.992
**Oscillibacter**	0.28 ± 0.29	0.25 ± 0.21	-0.082	0.738	0.992
**Lactobacillus**	0.5 ± 2.61	0.56 ± 2.17	0.831	0.755	0.992
**Anaerostipes**	1.39 ± 1.77	1.30 ± 1.40	-0.071	0.782	0.992
**Odoribacter**	0.06 ± 0.13	0.05 ± 0.05	0.083	0.783	0.992
**Bilophila**	0.06 ± 0.08	0.06 ± 0.07	-0.078	0.827	0.992
**Gordonibacter**	0.007 ± 0.02	0.009 ± 0.02	-0.116	0.83	0.992
**unclassified Porphyromonadaceae**	0.26 ± 0.727	0.19 ± 0.633	-0.314	0.852	0.992
**Desulfovibrio**	0.10 ± 0.21	0.17 ± 0.31	-0.149	0.859	0.992
**Veillonella**	0.05 ± 0.13	0.06 ± 0.15	0.172	0.864	0.992
**Clostridium_XlVa**	1.18 ± 1.77	1.43 ± 1.73	-0.035	0.87	0.992
**unclassified Veillonellaceae**	0.02 ± 0.04	0.03 ± 0.11	-0.176	0.893	0.992
**Catenibacterium**	0.47 ± 1.40	0.61 ± 1.52	0.467	0.894	0.992
**Alloprevotella**	0.22 ± 0.73	0.08 ± 0.23	-0.404	0.901	0.992
**Blautia**	6.65 ± 5.24	7.05 ± 5.77	0.015	0.913	0.992
**Paraprevotella**	0.15 ± 0.53	0.13 ± 0.31	-0.16	0.922	0.992
**Prevotella**	6.00 ± 11.16	5.6 ± 11.09	-0.362	0.926	0.992
**unclassified Prevotellaceae**	0.15 ± 0.80	0.08 ± 0.18	0.16	0.937	0.992
**Olsenella**	0.07 ± 0.38	0.03 ± 0.07	0.126	0.937	0.992
**Dialister**	1.02 ± 1.79	1.06 ± 1.74	0.149	0.959	0.992
**Bacteroides**	5.70 ± 6.78	6.73 ± 7.68	0.013	0.962	0.992
**Escherichia/Shigella**	1.00 ± 4.85	1.25 ± 5.97	-0.083	0.98	0.992
**Phascolarctobacterium**	0.55 ± 0.79	0.52 ± 0.92	0.027	0.985	0.992
**Streptococcus**	1.15 ± 2.75	1.23 ± 3.45	0.004	0.992	0.992

Subjects With Obesity					
**Genus**	**MHO (%)**	**MUO (%)**	**Log2FC**	**P value**	**Adjusted p value**
**unclassified Coriobacteriaceae**	0.25 ± 0.30	0.23 ± 0.57	-0.773	**0.02**	0.61
**Blautia**	7.01 ± 4.48	7.11 ± 5.98	-0.397	**0.023**	0.61
**Odoribacter**	0.06 ± 0.07	0.04 ± 0.05	-0.811	**0.034**	0.61
**Enterorhabdus**	0.03 ± 0.05	0.02 ± 0.03	-1.644	**0.041**	0.61
**Dorea**	1.41 ± 1.05	1.34 ± 1.03	-0.395	0.05	0.61
**Bifidobacterium**	2.65 ± 3.70	2.02 ± 3.41	-0.744	0.058	0.61
**Roseburia**	5.82 ± 4.70	7.92 ± 6.32	0.426	0.061	0.61
**Gemmiger**	1.56 ± 1.92	1.55 ± 1.72	-0.516	0.083	0.61
**Howardella**	0.05 ± 0.06	0.04 ± 0.05	-0.94	0.085	0.61
**Asaccharobacter**	0.04 ± 0.05	0.03 ± 0.05	-0.741	0.089	0.61
**Slackia**	0.05 ± 0.08	0.03 ± 0.05	-0.885	0.099	0.61

**Anaerofilum**	0.006 ± 0.009	0.005 ± 0.009	-0.852	0.102	0.61
**Alistipes**	1.22 ± 1.68	1.03 ± 1.34	-0.56	0.112	0.621
**Mogibacterium**	0.05 ± 0.07	0.04 ± 0.07	-0.857	0.144	0.712
**Allisonella**	0.019 ± 0.04	0.01 ± 0.02	-1.239	0.148	0.712
**Barnesiella**	0.42 ± 0.50	0.34 ± 0.71	-1.577	0.16	0.72
**Coprococcus**	3.61 ± 3.78	2.87 ± 2.58	-0.363	0.181	0.743
**Flavonifractor**	0.13 ± 0.15	0.11 ± 0.12	-0.501	0.186	0.743
**unclassified Lachnospiraceae**	9.33 ± 4.05	10.11 ± 4.45	-0.137	0.201	0.76
**Collinsella**	0.84 ± 1.07	0.75 ± 0.98	-0.342	0.247	0.887
**Gordonibacter**	0.008 ± 0.01	0.006 ± 0.01	-0.642	0.267	0.915
**unclassified Ruminococcaceae**	11.53 ± 8.14	12.22 ± 7.70	-0.217	0.315	0.984
**Anaerotruncus**	0.01 ± 0.02	0.017 ± 0.03	0.453	0.316	0.984
**Anaerostipes**	0.96 ± 0.95	0.85 ± 0.96	-0.293	0.338	0.984
**Peptococcus**	0.03 ± 0.06	0.03 ± 0.09	-0.835	0.355	0.984
**Clostridium_XlVa**	0.84 ± 0.76	1.19 ± 1.21	0.177	0.355	0.984
**unclassified Peptostreptococcaceae**	0.008 ± 0.02	0.004 ± 0.01	-0.801	0.378	0.984
**unclassified Erysipelotrichaceae**	0.90 ± 1.31	0.7 ± 1.11	-0.419	0.383	0.984
**Butyricimonas**	0.05 ± 0.06	0.05 ± 0.08	-0.425	0.413	0.99
**Bilophila**	0.07 ± 0.1	0.06 ± 0.08	-0.323	0.444	0.99
**Holdemania**	0.01 ± 0.02	0.01 ± 0.02	0.293	0.488	0.99
**Sporobacter**	0.005 ± 0.01	0.004 ± 0.01	-0.388	0.5	0.99
**Ruminococcus2**	0.92 ± 0.83	1.02 ± 1.52	0.17	0.501	0.99
**unclassified Veillonellaceae**	0.008 ± 0.02	0.009 ± 0.03	-0.634	0.51	0.99
**unclassified Desulfovibrionaceae**	0.04 ± 0.122	0.028 ± 0.12	-0.676	0.514	0.99
**Veillonella**	0.03 ± 0.05	0.07 ± 0.17	0.433	0.516	0.99
**Clostridium_XlVb**	0.15 ± 0.27	0.14 ± 0.17	-0.231	0.558	0.99
**unclassified Prevotellaceae**	0.25 ± 0.73	0.14 ± 0.77	-1.119	0.568	0.99
**Faecalibacterium**	11.26 ± 7.91	11.21 ± 7.91	0.13	0.608	0.99
**Ruminococcus**	2.24 ± 2.45	2.48 ± 2.32	0.124	0.656	0.99
**Desulfovibrio**	0.08 ± 0.23	0.10 ± 0.20	0.465	0.657	0.99
**Clostridium_XI**	1.94 ± 2.24	1.98 ± 2.61	0.149	0.683	0.99
**Turicibacter**	0.14 ± 0.29	0.12 ± 0.19	-0.215	0.688	0.99
**Butyricicoccus**	0.22 ± 0.19	0.2 ± 0.15	-0.084	0.722	0.99
**Haemophilus**	0.02 ± 0.04	0.06 ± 0.24	0.474	0.744	0.99
**unclassified Porphyromonadaceae**	0.19 ± 0.51	0.09 ± 0.20	0.459	0.768	0.99
**Akkermansia**	1.58 ± 3.96	1.23 ± 2.45	-0.728	0.78	0.99
**Bacteroides**	4.83 ± 5.45	5.56 ± 6.40	0.084	0.787	0.99
**Actinomyces**	0.02 ± 0.02	0.02 ± 0.03	-0.103	0.81	0.99
**Sutterella**	0.16 ± 0.40	0.05 ± 0.08	-0.318	0.819	0.99
**Olsenella**	0.09 ± 0.32	0.03 ± 0.06	-0.391	0.824	0.99
**Catenibacterium**	0.75 ± 1.88	0.78 ± 2.09	-0.689	0.832	0.99
**Dialister**	1.47 ± 2.47	0.67 ± 1.26	-0.675	0.835	0.99
**Rothia**	0.004 ± 0.01	0.01 ± 0.02	0.126	0.847	0.99
**Clostridium_sensu_stricto**	0.92 ± 1.29	0.95 ± 1.45	-0.08	0.853	0.99

**Prevotella**	8.73 ± 14.64	6.16 ± 12.04	-0.58	0.858	0.99
**Parabacteroides**	0.17 ± 0.22	0.19 ± 0.24	-0.073	0.87	0.99
**Paraprevotella**	0.05 ± 0.14	0.08 ± 0.21	0.262	0.878	0.99
**Alloprevotella**	0.14 ± 0.47	0.10 ± 0.26	-0.491	0.88	0.99
**Phascolarctobacterium**	0.40 ± 0.61	0.45 ± 0.55	-0.196	0.882	0.99
**Victivallis**	0.015 ± 0.07	0.005 ± 0.02	-0.213	0.886	0.99
**Escherichia/Shigella**	1.18 ± 4.15	1.49 ± 6.65	0.398	0.902	0.99
**Eggerthella**	0.009 ± 0.02	0.015 ± 0.06	-0.124	0.903	0.99
**Parasutterella**	0.07 ± 0.21	0.07 ± 0.14	0.074	0.922	0.99
**Clostridium_XVIII**	0.26 ± 0.56	0.21 ± 0.33	-0.039	0.928	0.99
**Streptococcus**	2.19 ± 6.74	1.69 ± 2.99	0.079	0.961	0.99
**Methanobrevibacter**	0.07 ± 0.16	0.07 ± 0.19	0.056	0.967	0.99
**Lactobacillus**	0.26 ± 0.81	1.69 ± 6.06	0.131	0.968	0.99
**unclassified Oxalobacteraceae**	0.004 ± 0.009	0.004 ± 0.01	0.024	0.978	0.99
**Lachnospira**	0.69 ± 0.64	0.71 ± 0.93	0.009	0.979	0.99
**Oscillibacter**	0.22 ± 0.16	0.26 ± 0.33	-0.006	0.984	0.99
**Clostridium_IV**	0.77 ± 1.27	1.16 ± 2.28	-0.004	0.99	0.99

P values < 0.05 are in bold.

Adjusted p values were calculated by DESeq2 test with Benjamini–Hochberg correction.

Permutational multivariate analysis adjusted for age, sex, disease status (CVD and T2DM), medication use (blood pressure, cholesterol lowering and diabetes medication), lifestyle factors (DASH score, smoking status and alcohol consumption).

Table S6 Significant results of linear regression between metabolic health markers and phylum relative abundance

Health markers	Genus	Coefficient	R^2	p value	FDR
**Glucose**	Verrucomicrobia	-0.612	0.021	**<0.001**	**0.009**

P values < 0.05 are in bold. Only significant results are shown.
Permutational multivariate analysis adjusted for age, sex, disease status (CVD and T2DM), medication use (blood pressure, cholesterol lowering and diabetes medication), lifestyle factors (DASH score, smoking status and alcohol consumption).

Table S7 Significant results of linear regression between metabolic health markers and family relative abundance

Health markers	Genus	Coefficient	R^2	p value	FDR
**Fat percent**	Acidaminococcaceae	-0.024	0.032	**0.001**	**0.010**
**BMI**	Clostridiales_Incertae_Sedis_XIII	0.002	0.065	**0.008**	**0.047**
**Lowest Rib Circumference**	Acidaminococcaceae	0.001	0.068	**0.005**	**0.047**
**Fat percent**	Clostridiales_Incertae_Sedis_XIII	0.001	0.064	**0.012**	**0.047**
**Fat percent**	Pasteurellaceae	-0.007	0.023	**0.004**	**0.047**
**Glucose**	Verrucomicrobiaceae	-0.608	0.021	**0.001**	**0.009**

P values < 0.05 are in bold. Only significant results are shown.
Permutational multivariate analysis adjusted for age, sex, disease status (CVD and T2DM), medication use (blood pressure, cholesterol lowering and diabetes medication), lifestyle factors (DASH score, smoking status and alcohol consumption).

Table S8 Significant results of linear regression between metabolic health markers and genus relative abundance

Health markers	Genus	Coefficient	R^2	p value	FDR
**HDL-C**	Blautia	2.84E+00	0.050	**<0.001**	**0.003**
**Glucose**	Akkermansia	-6.08E-01	0.021	**<0.001**	**0.009**
**Waist circumference**	Gemmiger	2.27E-02	0.030	**<0.001**	**0.007**
**BMI**	Anaerostipes	-5.45E-02	0.067	**0.002**	**0.019**
**Waist circumference**	Anaerostipes	-1.80E-02	0.061	**0.005**	**0.031**
**Total cholesterol**	Clostridium_IV	2.29E-01	0.052	**0.003**	**0.018**
**LDL-C**	Clostridium_IV	2.71E-01	0.055	**0.002**	**0.018**
**Triglyceride**	Collinsella	2.20E-01	0.054	**0.002**	**0.015**
**VLDL-C**	Collinsella	5.10E-01	0.056	**0.001**	**0.015**
**Fat percent**	Phascolarctobacterium	-2.18E-02	0.032	**0.001**	**0.017**
**DBP**	Parabacteroides	4.88E-03	0.031	**0.003**	**0.039**
**HbA1C**	Paraprevotella	1.06E-02	0.029	**0.003**	**0.034**
**BMI**	Mogibacterium	1.83E-03	0.065	**0.008**	**0.047**
**Waist circumference**	Mogibacterium	7.29E-04	0.068	**0.005**	**0.047**
**Fat percent**	Mogibacterium	1.35E-03	0.064	**0.012**	**0.047**
**SBP**	Allisonella	8.85E-04	0.031	**0.003**	**0.034**
**BMI**	Anaerofilum	2.39E-04	0.034	**0.010**	**0.023**
**Waist circumference**	Anaerofilum	1.05E-04	0.041	**0.002**	**0.009**
**Triglyceride**	Anaerofilum	2.01E-03	0.040	**0.002**	**0.009**
**Fat percent**	Anaerofilum	2.15E-04	0.039	**0.002**	**0.009**
**VLDL-C**	Anaerofilum	4.29E-03	0.038	**0.003**	**0.009**

P values < 0.05 are in bold. Only significant results are shown.

Permutational multivariate analysis adjusted for age, sex, disease status (CVD and T2DM), medication use (blood pressure, cholesterol lowering and diabetes medication), lifestyle factors (DASH score, smoking status and alcohol consumption).
